# Human papilloma virus infection and its associated risk for cervical lesions: a cross-sectional study in Putuo area of Shanghai, China

**DOI:** 10.1186/s12905-023-02166-w

**Published:** 2023-01-20

**Authors:** Haiping Luan

**Affiliations:** Department of Laboratory, Putuo District Maternal and Child Health Hospital, No. 517 of Tongpu Street, Putuo District, Shanghai, 200062 China

**Keywords:** HPV, Cervical cancer, Screening

## Abstract

**Objective:**

To investigate the human papilloma virus (HPV) infection status, main subtypes and age distribution characteristics of women in the Putuo area of Shanghai.

**Methods:**

A total of 13,936 subjects were enrolled in this study. These subjects were 15–89 years old, with a mean age of 41.68. Real-time fluorescence quantitative polymerase chain reaction technology was used to detect 21 types of HPV.

**Results:**

A total of 2,500 subjects with HPV infections were detected in 13,936 cervical exfoliated cell specimens (total infection rate 17.9%). There were 15 people aged below 20,486 people aged 21-30,876 people aged 31-40,484 people aged 41–50, 338 people aged 51–60, and 301 people aged > 60. In total, 1,893 (75.7%) subjects had a single type of HPV infection, 424 (16.9%) had a double infection, and 183 had triple or more infections (7.4%). The top 6 subtypes of HPV infection in the Shanghai Putuo District were HPV 52 (3.81%), HPV 58 (2.46%), HPV 16 (2.43%), HPV 53 (2.30%), HPV 81 (1.74%) and HPV 39 (1.5%). The number of high-risk HPV infections was 1,978, and the total infection rate was 14.19%. The number of intermediate-risk HPV infections was 578, and the total infection rate was 4.15%. The number of low-risk HPV infections was 338, and the total infection rate was 2.43%.

**Conclusion:**

The top 3 populations with HPV infection rates in the Putuo District, Shanghai, were ≤ 20 years old, older than 60, and 21–30 years old. The infection rate of HPV in cervical outpatient clinics was significantly higher than that of other departments. The 9-valent vaccine is recommended for HPV vaccination in this area.

## Introduction

Human papilloma virus (HPV), belonging to the papilloma vacuolar virus, is a small covalent double-stranded circular DNA virus mainly present in human skin, mucosa and female cervical epithelial cells. More than 200 kinds of HPV have been found [1–3]. Studies have shown that long-term repeated infection of certain subtypes of HPV is a risk factor for cervical cancer development in women. According to the World Health Organization (WHO), there are about 500,000 new cervical cancer cases annually. Cervical cancer is a common malignant tumour in the female reproductive tract and is ranked fourth in cancer mortality rate and second in female cancer [4–7]. About 80,000 women in China die from cervical cancer every year, so screening for HPV is necessary. In the face of these public health challenges, it is necessary to strengthen the management of medical resources and build a comprehensive system plan to promote the preparedness of communities and hospitals for diseases, so as to effectively curb the spread of these diseases [8–10].

At present, laboratory testing methods mainly include nucleic acid testing, cytology testing, pathological histology testing, and so on. The current HPV vaccination campaign is also in full swing. Some experts also believe that the HPV subtype infection situation and the region’s age factors should be considered for HPV vaccination. Among them, the HPV high-risk type tends to cause intraepithelial neoplasia of cervical cells. In contrast, the HPV low-risk type of infection causes genital tract warts. The HPV high-risk types include HPV 16, HPV 18, HPV 31, HPV 33, HPV 35, HPV 39, HPV 45, HPV 51, HPV 52, HPV 56, HPV 58, HPV 59, and HPV 68, five intermediate-risk types including HPV 26, HPV 66, HPV 53, HPV 73, and HPV 82, and three low-risk types including HPV 6, HPV 11, and HPV 81 [11]. However, there remains uncertainty regarding the prevalence of HPV infection and cervical risk in China. It is necessary to provide clinical evidence for the prevention of HPV infection and cervical cancer.

In this study, with patients in the department of gynaecology, the cervical department, the obstetrical department, and the physical examination department in the Putuo District, Shanghai, as study subjects, 21 subtypes of HPV infection were tested, using real-time quantitative polymerase chain reaction (Polymerase Chain Reaction, PCR) technology, aimed to understand the epidemiological characteristics of HPV in women in Shanghai in detail, analyse and evaluate the pathogenic risk of cervical lesions of different high-risk or suspected high-risk HPV, and provide a reference for developing cervical cancer screening strategies and the research, development and distribution of HPV vaccines suitable for China's national conditions.

## Subjects and methods

### Study design and subjects

This study is a retrospective, single-center study. A total of 13,936 females were selected to voluntarily undergo HPV examination in the departments of gynaecology, cervical, obstetrical and physical examination of the Shanghai Putuo District Maternal and Child Health Hospital from June 2021 to June 2022.

### Research methods

#### Sample collection

The study subjects met the requirements: (1) the non-menstrual period; (2) Intravaginal irrigation and medication should not be performed 24 h before sampling. Cervical exfoliated cells were collected by routine gynaecological examination with a vaginal speculum to enlarge the vagina and to rotate the cervical sampler brush clockwise or counter clockwise for three to four laps. After sampling, it was placed in the storage solution of cervical exfoliated cell samples and sent to the specimen receiving laboratory.

#### Instruments and reagents

For HPV testing, an HPV typing testing kit (Jiangsu Shuoshi Biotechnology Co., Ltd., Taizhou, China, JC80301) was used. The SLAN-96P fluorescence quantitative PCR instrument (Shanghai Hongshi Medical Technology Co., LTD., Shanghai, China) was used for PCR analysis. HPV detection and typing method: Real-time quantitative polymerase chain reaction (Polymerase Chain Reaction, PCR) technology was used to target the human papillomavirus genome L1 region, 21 subform-specific primers and probes were designed, and the corresponding subtypes were marked with FAM, HEX and ROX, respectively. The probes were oligonucleotides, including the 5′-end reporter dye and the 3′-end quencher dye. During PCR amplification, when the probe is complete, the fluorescence emitted from the reporter dye is absorbed by the quencher dye due to its proximity to the reporter dye and emits no fluorescence signal. When the primer was extended, the probe bound to the template was cut off by the Taq enzyme (5′ → 3′ exonuclease activity). The reporter dye was separated from the quencher dye to produce the fluorescence signal. The quantitative PCR instrument automatically drew the real-time amplification curve according to the detected fluorescence signal to realise the qualitative detection of human papillomavirus on the nucleic acid level. At the same time, reference genes can be used to monitor and exclude false negatives due to instrument failure, reagent factors, improper manipulation, or inhibitors in the sample. The instrument automatically saves the results after the reaction. The baseline start point, endpoint, and threshold can be adjusted according to the analysed image. The result can also be automatically read by the instrument (default start cycle 6, stop cycle 12, and threshold 0.12). Click Analysis to automatically obtain the analysis results and find the results in the same interface.

#### Quality control

Positive plasmids and blank controls were used to monitor the amplification reactions during the amplification process. The experiment participated in the Shanghai temporary inspection centre room quality control as a laboratory quality assurance. Blank control: No typical S-type amplification curve is shown. Positive control: the HPV 21 subtypes and the reference gene test showed a typical S-type amplification curve and a CT value ≤ 30. Reference gene: The FAM channel amplification curve in group H has a typical S-type curve and CT ≤ 36.7, otherwise, it is related to errors caused by sampling, transportation, preservation conditions and experimental operation. The above requirements should be met simultaneously in the same experiment. Otherwise, the qualitative results of this experiment were invalid.

#### Statistical methods

Statistical analysis was performed using SPSS17.0 (IBM. Corp, Silicon Valley, CA, USA). Measurement data were tested for normality using the Shapiro–Wilk test. Data following normal distribution were described by the mean ± standard deviation. The count data were statistically described by number (%). The rate of HPV infection was compared using the chi-square test. *P* < 0.05 was considered a statistically significant difference. The sample size was calculated using the PASS software (version 15, NCSS, LLC. Kaysville, Utah, USA, ncss.com/software/pass) with the module of confidence interval for one proportion. A sample size of 715 produces a two-sided 95% confidence interval with a width equal to 0.060 when the sample proportion is 0.200.

## Results

### General data and HPV infection status of the study subjects

A total of 13,936 study subjects were included, aged 15–89, with a mean age of 41.7 ± 12.3 years. Subjects aged 31–40 accounted for the highest proportion (36.17%). All patients were from urban areas and had experienced sexual activity but were not HPV vaccinated. Most patients were married (89.16%) and were educated to the level of junior high school or above (82.71%). In addition to the presence or absence of HPV infection, some patients were infected with other sexually transmitted diseases, such as Ureaplasma urealyticum (47.93%) (Table 1).

**Table 1:** General demographic characteristics of the study subjects

Variable	Number	*P* value
*Age (years old)*		
≤ 20 years old	45 (0.32%)	< 0.001
21–30 years old	2542 (18.24%)	
31–40 years old	5041 (36.17%)	
41–50 years old	3066 (22.00%)	
51–60 years old	1788 (12.83%)	
> 60 years old	1454 (10.43%)	
*Habitation*		
Rural area	0	< 0.001
Town	13,936 (100%)	
*Education*		
Junior high school and below	2410 (17.29%)	< 0.001
Junior high school and above	11,526 (82.71%)	
*Marriage*		
Unmarried	1278 (9.17%)	< 0.001
Married	12,426 (89.16%)	
Divorce or widowed	232 (1.66%)	
*Virgin or not*		
Yes	0	< 0.001
No	13,936 (100%)	
*Occupation*		
Unemployed	2584 (18.54%)	< 0.001
Employed	11,352 (81.46%)	
*Procreation status*		
Unbearing	4947 (35.49%)	< 0.01
Preprocreated	8989 (64.50%)	
*HPV vaccination status*		
Yes	0	< 0.001
No	13,936 (100%)	
*Other sexually transmitted diseases*		
Chlamydia trachomatis	735 (5.27%)	< 0.05
Neisseria gonorrhoeae	5 (0.03%)	
Ureaplasma Urealyticum	6680 (47.93%)	

*HPV*: human papilloma virus

### Infection status of HPV-positive patients

A total of 2,500 HPV infections were detected in 13,936 HPV screening specimens, and the total positive rate was 17.9%, among which the highest positive rate was respectively: HPV 52 (3.81%), HPV 58 (2.46%), HPV 16 (2.43%), HPV 53 (2.30%), HPV 81 (1.74%) and HPV 39 (1.52) (Table 2).

**Table 2:** Infection of different HPV subtypes

HPV subtype	The number of detection	Infection rate of total subjects (%)	Infection rate of HPV infected subjects (%)
HPV52	531	3.81	21.24
HPV58	343	2.46	13.72
HPV16	339	2.43	13.56
HPV53	321	2.30	12.84
HPV81	243	1.74	9.72
HPV39	212	1.52	8.48
HPV66	175	1.26	7.00
HPV56	159	1.14	6.36
HPV68	153	1.10	6.12
HPV51	136	0.98	5.44
HPV31	131	0.94	5.24
HPV59	127	0.91	5.08
HPV18	126	0.90	5.04
HPV33	114	0.82	4.56
HPV35	74	0.53	2.96
HPV6	56	0.40	2.24
HPV82	39	0.28	1.56
HPV11	39	0.28	1.56
HPV73	31	0.22	1.24
HPV45	26	0.19	1.04
HPV26	12	0.09	0.48

HPV: human papilloma virus

### Comparison of HPV detection among all ages

A total of 2,500 individuals with HPV infection were found among the 13,936 cervical detached cell specimens, for an overall infection rate of 17.9%. The 13,936 patients were classified into six age groups: patients under the age of 20, patients between the ages of 21 and 30, patients between the ages of 31 and 40, patients between the ages of 41 and 50, patients between the ages of 51 and 60, and patients older than 60. Using quantitative PCR, the infected population was segmented into 15 patients under the age of 20 (infection rate: 33.33%), 486 patients between the ages of 21 and 30, 876 patients between the ages of 31 and 40, 484 patients between the ages of 41 and 50, 338 patients between the ages of 51 and 60, and 301 patients over the age of 60 (infection rate: 20.70%). The infection rates among the age categories varied significantly (Table 3).

**Table 3:** Analysis of HPV infection in each age group

Age	Infection rate	*χ2* value	*P* value
≤ 20 years old	15/45 (33.33%)	29.05	< 0.001
21–30 years old	486/2542 (19.11%)		
31–40 years old	876/5041 (17.38%)		
41–50 years old	484/3066 (15.79%)		
51–60 years old	338/1788 (18.90%)		
> 60 years old	301/1454 (20.70%)		

HPV: human papilloma virus

### Analysis of infection with different HPV subtypes

**Fig. 1:**
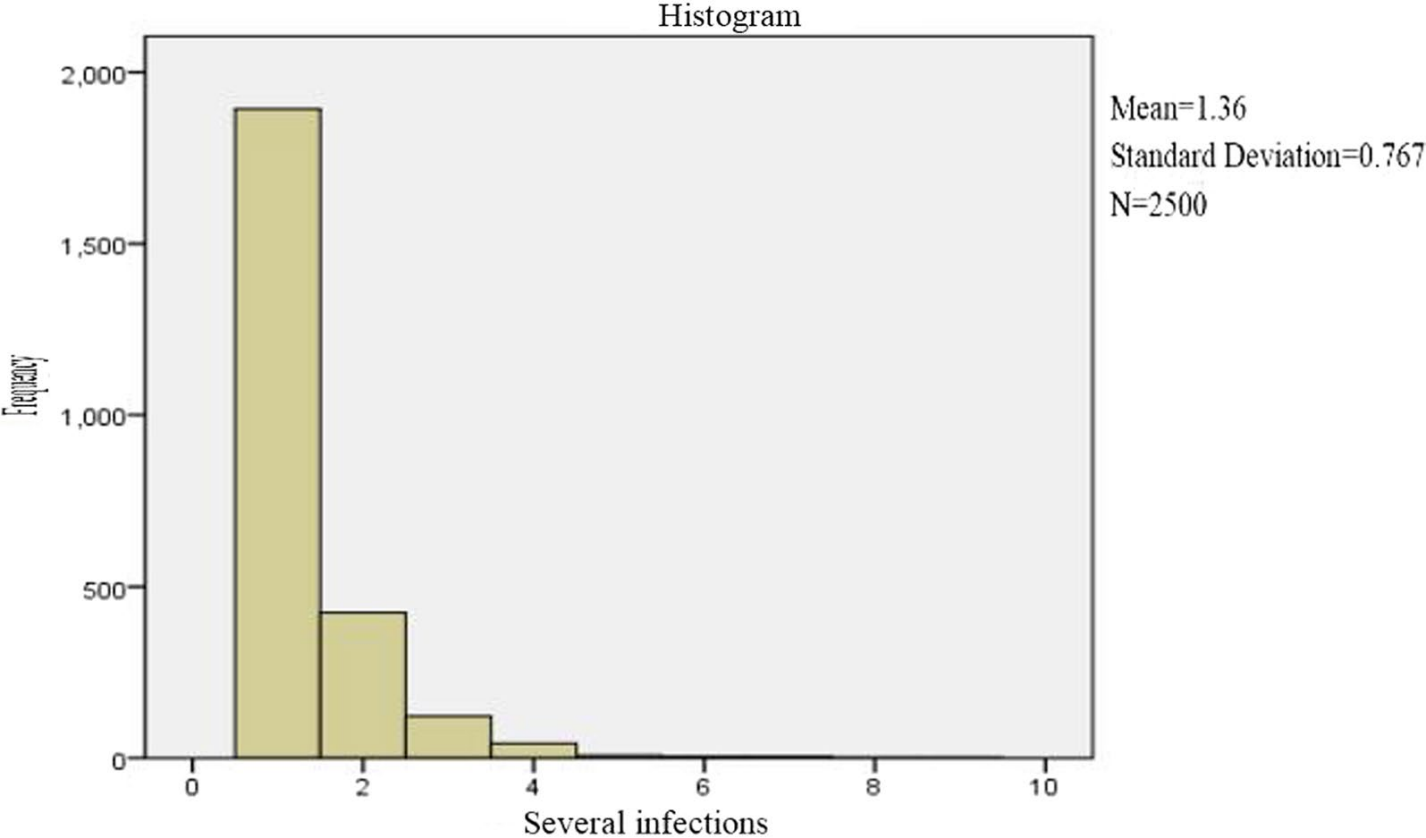
Distribution of several heavy infections among 2500 HPV infections

**Fig. 2:**
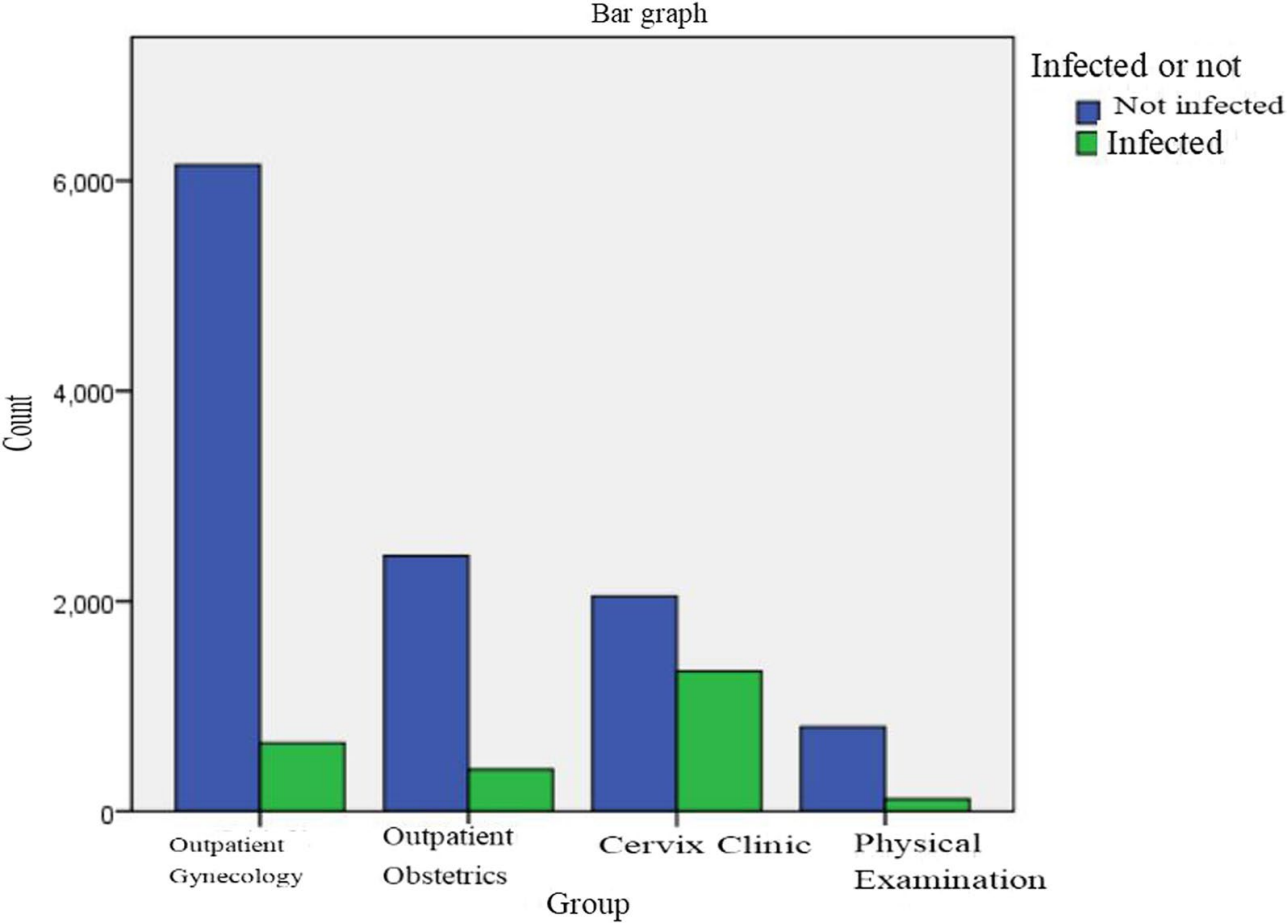
Distribution of different departments in 2500 cases of HPV infection

Of the 2,500 HPV-positive specimens, 1,893 (75.7%) were single infections, 424 (16.9%) were double infections, and 183 (7.4%) were triple or more infections (Fig. 1). For 2,500 HPV-positive specimens, the number of infections was 399 (14.08%) in the outpatient obstetric department, 1,334 (39.46%) in the outpatient cervical department, 650 (9.55%) in the outpatient department of gynaecology and 117 (12.72%) in the outpatient physical examination department (Fig. 2). There were statistically significant differences among the various departments (*X*^2^ = 1433.6, *P* < 0.05). Of all the 13,936 specimens, the numbers of high-risk, moderate risk, and low-risk HPV infections were 1,978 (14.19%), 578 (4.15%), and 338 (2.43%), respectively.

## Discussion

### HPV infection and age showed a "V" type distribution

This study shows that the top three ages with a high incidence of HPV are ≤ 20 years old, > 60 years old and 21–30 years old. Therefore, we should not only vaccinate these people with the HPV vaccine in advance but also pay attention to the importance of reproductive health and HPV vaccination in our daily science popularisation work. Investigating the prevalence of HPV infection according to different age populations is of great importance to achieve the precision prevention and establish appropriate therapeutic strategies [12, 13]. For women aged > 60 years, the HPV infection rate is also high, so different management for HPV is targeted at different populations. Women aged > 60 can be diagnosed primarily with HPV screening combined with thin-cytologic test (TCT) results.

### HPV virus infection rate and subtype infection situation

Our findings provided further insights into the prevalence of HPV infections according to different subtypes, which was useful to establish the epidemiological distribution pattern of HPV in the local area and provide guidance for secondary and tertiary prevention. In this study, the total infection rate of 13,936 women was 17.9%, lower than the 22.6% reported in the Beijing area [14] and lower than the 26.26% reported in Shaanxi province [15]. This finding indicated that the distribution pattern of HPV infection might exhibit specific characteristics from different regions and living habits. The top three subtypes of HPV infection in the Putuo District were HPV 52 (3.8%), HPV 58 (2.5), and HPV 16 (2.4%), which are consistent with the top three subtypes reported by Ming Chunyan et al. [16]. These subtypes of HPV infections might be the focus of prevention and treatment of cervical cancer in the future. Previous studies have shown that persistent infection of the HPV 16 subtype is closely related to cervical cancer, and HPV 16, HPV 52, and HPV 58 cause specific cancer in Chinese women, so secondary prevention against cervical cancer is still essential in regional screening [17–19]. Because chronic cervicitis leads to cervical cancer under chronic HPV virus repeated infection, long-term follow-up is still necessary [20, 21].

HPV 52 had a higher incidence of cervical lesions compared to HPV 16, HPV 53, and HPV 58. The findings suggested that HPV 56, HPV 58, and HPV 52 were associated with an increased incidence of cervical lesions compared to HPV 16, and that HPV 53/58/52 infection individuals may also be sent for colposcopy in addition to HPV 16 and 18. There are few related studies on other HR-HPV cancer-causing forces, and no unified cognition has been formed. Wang et al. [22] reported a higher detection rate of HSIL in HPV 16/18/31/33/52/58 infected persons after colposcopy. The conclusions of Sung et al. [23] also suggest that more aggressive action may be needed for HPV 52/58-infected patients.

The present study has some research limitations. First, the source of our sample is relatively limited, only for population screening in key departments, and cannot represent the screening results of the whole population, which may have some bias in the extrapolation of the results. Second, we have only analysed the infection status of HPV and have not yet focused on the relationship between HPV infection and its outcome shunt, which should be further deepened in future studies. Third, the generalizability of our results is limited due to our single-center design. The validity of this study would increase if the rural population was also included. Finally, we have not analysed the association between individual basic characteristics and HPV infection, and therefore, we will analyse the association in more depth in subsequent studies.

## Conclusion

In conclusion, HPV infections in the Putuo area, Shanghai, is still at a high level, and the top-ranked HPV infection subtypes are HPV 52, HPV 58, HPV 16, and HPV 53. We should take more proactive measures against HPV 52/58/53 infection with the cervical cancer screening strategy, vaginal referral, and the future development of the HPV vaccine in China, as it serves as a reminder that the HPV vaccine vaccination is still the primary method of preventing and controlling cervical cancer at our primary level. Further studies are warranted to elucidate the mechanism behind the association with HPV infection and risk of cervical lesions in this area with a long-term follow-up.

## Data Availability

All data generated or analyzed during this study are included in this article.
